# Phytochemical Investigation on Volatile Compositions and Methoxylated Flavonoids of *Agrostis gigantea* Roth

**DOI:** 10.22037/ijpr.2019.15209.12935

**Published:** 2020

**Authors:** Mahmoud Rafieian-kopaei, Azadeh Hamedi, Ebrahim Soleiman Dehkordi, Arsalan Pasdaran, Aradalan Pasdaran

**Affiliations:** a *Medicinal Plants Research Center, Basic Health Sciences Institute, Shahrekord University of Medical Sciences, Shahrekord, Iran. *; b *Department of Pharmacognosy, School of Pharmacy, Shiraz University of Medical Sciences, Shiraz, Iran. *; c *Medicinal Plants Processing Research Center, Shiraz University of Medical Sciences, Shiraz, Iran. *; d *Senior Researcher, Kara Daru & Revive Chemistry Co. Shiraz, Iran.*

**Keywords:** Methoxylated flavonoids, Agrostis gigantea Roth, 4T1 breast carcinoma cell, Aryl hydrocarbon receptor

## Abstract

In this study, methoxylated flavonoids and volatile constitutions of *Agrostis gigantea* Roth (Poaceae) were investigated for the first time. The flavonoids were identified by spectroscopic methods (^1^H-NMR, ^13^C-NMR, COSY, NOSEY, TCOSY, and HMBC). The volatile constitutions of aerial parts and seeds were analyzed by gas chromatography–mass spectrometry (GC-MS). Two methoxylated flavonoids, luteolin 5-methyl ether (1), and cirsilineol (2) were isolated from the aerial parts of this plant. According to the GC-MS data the main constitutions of these volatile oils belong to the simple phenolic category which include coniferyl alcohol (18.80%) and eugenol (12.19%) in aerial parts and seeds, respectively. By using the computer- aided molecular modeling approaches, the binding affinity of these compounds was predicted in the catalytic domains of aryl hydrocarbon receptor (AhR).

These two isolated flavonoids were investigated *in-vitro* for their inhibitory activity on 4T1 breast carcinoma cells. It was predicted that these compounds could be well-matched in aryl hydrocarbon receptor (3H82) active site, but based on the *in-vitro* assay, the IC_50_ values on cytotoxicity were 428.24 ±3.21 and 412.7±3.02 μg/mL for luteolin 5-methyl ether and cirsilineol, respectively. Thus, it can be concluded that these flavonoids exhibit low cytotoxicity against 4T1 breast carcinoma cell line.

## Introduction

Numerous laboratory studies have demonstrated that flavonoids rich diets could exhibit protection and inhibition effects on carcinogens(1) . This potential has been observed in several steps of cancer initiation and development stages, for example, inhibition of various endogenous or exogenous carcinogen activation steps such as CYP1A1 enzyme activation (2). From pharmacological viewpoint, it has been shown that the main mechanism of action of this class of compounds is agonistic or antagonistic effects on aryl hydrocarbon receptor (AhR) (3-5). The inhibition of AhR-mediated signal transduction pathway is generally considered as a valuable preventive and therapeutic mechanism in various cancers especially in breast cancer (6). At the promotion stage of cancer, a wide variety of mechanisms could be involved in the flavonoids positive effects on carcinogenic cells which include inactivation of epidermal growth factor receptor (EGFR), inhibition of thymidylate synthase, inhibition of thioredoxin reductase, acting on glucose-regulated protein 78, changing of the vascular endothelial growth factor (VEGF) and hypoxia-inducible factor 1 (HIF-1) expression (7-10). Studies have indicated that CYP1A1 and 1B1 are regulated by the ligand-activated aryl hydrocarbon receptor (AhR). Benzo[a]pyrene is well known as AhR activator (one of the most potent carcinogenesis pathway) (11, 12). Inhibition of the bio-activation of AhR-mediated signal transduction pathway has been demonstrated for some methoxylated flavonoids such as 3′, 4′-dimethoxyflavone, and 5, 7- dimethoxyflavone in previous studies. These compounds potentially inhibited BaP-induced CYP1A1 protein expression and other related AhR mediated pathways (13, 14). In addition to the prevention potential, antiproliferative properties of methoxylated flavonoids on cancer cells have also been demonstrated. These investigations have shown more apoptotic potential of methoxylated flavones than un-methoxylated flavones for various carcinogenic cells (15). The greater potency of this class of compounds versus the hydroxylated flavones could be attributed to greater cancer cell uptake of the methoxylated flavones (16). Therefore, flavonoids rich plants are considered as one of the main anticancer metabolites screening source. Poaceae or Gramineae is a well-known monocotyledonous plant which includes the most important edible plants such as wheat, rice, and many other cereals. *Agrostis gigantea *Roth “redtop” (Poaceae), redtop is a perennial invasive herb native to Eurasia and North Africa culms up to 4 feet in height with folded or flat leaf(17, 18). Nevertheless, redtop has been commonly used by livestock and birds for a long time but no research has been carried out on chemical compounds and biological potential of this plant until now. In this report, two methoxylated flavonoids, luteolin 5-methyl ether (**1**), and cirsilineol (**2**), were isolated from *A. gigantea* Roth “redtop” (Poaceae), and their structures were elucidated and also, their effects on 4T1 breast cancer cell line were investigated by *in-silico* and *in-vitro* methods.

## Experimental

*Plant material*

The aerial parts of *A. gigantea *were collected from Saravan region in Guilan province in May 2018. This plant was given a voucher specimen number (MPPRC-98-6) at the Medicinal Plants Processing Research Center, Shiraz University of Medical Sciences, Shiraz, Iran.

*Extraction and isolation of flavonoids*

**Figure 1:**
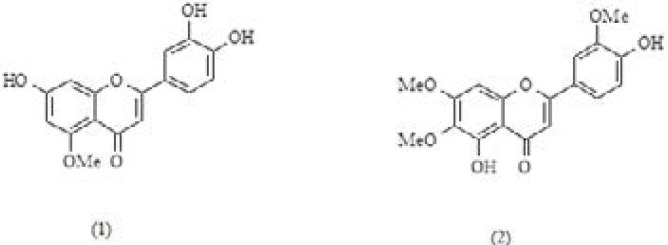
Luteolin 5-methyl ether (***1***) and cirsilineol (***2***) chemical structures isolated from *A. gigantea *aerial parts

The air-dried powdered aerial part of *A. gigantea *(2.5 kg) was extracted by maceration method with methanol (MeOH) (20 L× 3) at room temperature for three days. This extract was concentrated with rotary evaporator in vacuo condition at 45 °C. This methanolic extract (110 g) was dissolved in distilled water and thus was partitioned with *n*-hexane, dichloromethane (DCM), ethyl acetate (EtOAc), and butanol (BuOH) (2 L× 3 for each solvent). These fractions were dried using the rotary evaporator which yielded 11 g from hexane, 9.00 g from DCM, 8.5 g from EtOAc and 9.6 g from BuOH (Fraction A-D). Dichloromethane fraction (fraction D) (9.00 g) was chromatographed on silica gel column (5 × 100 cm, 1000 g silica gel, particle size: 60–200 μm) with a hexane: EtOAc step gradient (100:0, 80:20, 60:40, 40:60, 20:80, and 0:100). After the second fractionation, all fractions (fractions D_1_-D_6_) were analyzed using analytic silica gel thin layer chromatography, and the fractions with the same pattern were mixed together. Based on the TLC pattern, fractions D_4_, D_5_, D_6 _(totally, 362 mg) were mixed together and was subjected to Sephadex-LH20 column (5× 60 cm, 500 g of LH20 dispersed in methanol), using MeOH (3 L) as the mobile phase to give compound (**1**) (18.3 mg) and compound (***2***) (Figure 1**, **Table 1) (15.6 mg). NMR spectra of these compounds were collected in deuterated methanol at room temperature using an AVANCE AV-III HD Bruker 400 spectrometer (400 MHz for^ 1^H NMR). Deuterated methanol was used as a standard reference. The pulse programs for 2D experiments parameters were chosen from Bruker software library as follows: HSQC spectra were gathered based on 400/100 MHz with D_1_ =2.0 s relaxation delay time and D _21 _= 344 ms for delay for multiplicity selection; 400 MHz gradient-selected for TCOSYs spectra, recycling delay between two scans D_1_ =1.5 s and mixing time D_9_= 100 ms. 400/100 MHz gradient-selected HMBC spectra, relaxation delay D_1_ =2.0 s, evolution delay D_2_ = 3.44 ms, delay for evolution of long-range coupling D_6 _= 50 ms; 400 MHz gradient-selected ^1^H,^1^H COSY spectra, relaxation delay D_1_=1.5 s, 90° pulse for ^1^H; 400MHz NOESY spectra, relaxation delay D_1_ =1.5 s, mixing time D_8_ = 300 ms. 

*Extraction of the essential oils*

The aerial parts and seeds of *A. gigantea *were dried in the laboratory for 6 days at 25 °C. Moisture content of these samples was determined based on the Association of Official Agricultural Chemists (AOAC) method by calculating samples weight loss after drying using air-oven at 110 °C for 4 h (19-21). Then, air-dried aerial parts and seed of this plant were finely grounded into a powder weighing 500 g and subjected to hydrodistillation (HD) for 3 h using a Clevenger type apparatus, yielding 0.9, and 0.6% v/w (based on dry plant materials). These samples were dehydrated with Na_2_SO_4_ and stored at 4 °C in the dark until tested and analyzed using gas chromatography (GC) and gas chromatography–mass spectrometry (GC–MS). 

*Analysis of the essential oils*

The essential oils of the *A. gigantea *aerial parts and seeds were analyzed with Shimadzu GC-MS-QP5050A equipped with the fused methyl silicon DB-5 column (60 m × 0.25 mm i.d., 0.25 μm film thickness). Helium at a flow rate of 1.3 mL/min was used as the carrier gas. The column temperature was kept at 50 °C for 3 min, which increased to 300 °C at a rate of 5 °C/min and finally kept at 300 °C for 5 min. The injector temperature was controlled at 270 °C, and the split ratio was adjusted at 1:33. The injection volume was 1 µL. 70 eV ionization energy with an electron impact (EI) quadrupolar system was used for GC/MS detection. Ion source temperature was fixed at 200 °C, and quadrupole temperature at 100 °C. 2 min solvent delay was considered, and EM voltage was 3,000 volts. Other conditions were included: resolution of 2,000 amu/s, and an amu scan range of 30–600 amu (22-24). The chemical components were identified based on computer matching with the NIST NBS54K Library, mass spectra, and relative retention times with Kovats indices (25). For quantization (area %), GC analyses were performed on an Agilent 6890 series apparatus fitted with an FID detector. The FID detector’s temperature was 300 ºC. To obtain the same elution order as with GC–MS, a simultaneous auto-injection was performed on a duplicate of the same column applying the same operational conditions. Relative percentage amounts of the separated compounds were calculated from FID chromatograms.

*Molecular Modelling*

Online tools were used to identify some molecular properties which include a number of hydrogen bond donors and acceptors, polar surface area, log P, and others. These compounds had no violation score of more than zero. The energy of each compound was minimized with Swiss-PDB Viewer V.4.1.0. The final conformations were used as starting conformations to perform docking. Docking was performed with Auto Dock Vina (Scripps Research Institute, USA) using Lamarckian Genetic Algorithm, the grid box includes all functional amino acids which were chosen in the active site region (26). For each compound, receptors with a favorable free energy of binding (kcal/mol) and positional root-mean-square deviation (RMSD) < 2.0 Å were gathered and represented as results. Active site analysis of the AhR receptors was carried out using Pymol and Discovery studio visualizer software.

*Receptors preparation*

The three-dimensional structures of four aryl hydrocarbon receptors were obtained from Protein Database (PDB: IDs 5UFP, 5TBM, 4XT2, and 3H82). The crystalline structures of these selected enzymes with their inhibitors were downloaded from the Protein Data Bank RCSB PDB (27). These receptors were prepared for docking by removing water, ions, etc using Discovery studio 2016 client software.

*Cell culture*

To determine the effects of luteolin 5-methyl ether and cirsilineol, 4T1 cells were plated in 1:1 mixture of Dulbecco’s Modified Eagle’s Medium (DME) and Ham’s F-12 Nutrient Mixture (DMEM/F12) medium containing 100 mg/L of streptomycin, 100,000 U/L of penicillin and 100 mL/L of fetal bovine serum (FBS). Before treating cells with the compounds, these primary cultures were rinsed and starved for 24 h in DMEM/F12, 0.1 g/L of BSA, 5 µg/L of selenium, and 5 mg/L of transferrin. After 24 h serum starvation, the cells received serum free medium containing various concentrations (2-1000 μM) of luteolin 5-methyl ether or cirsilineol.

*Methyl thiazolyl tetrazolium (MTT) assay*

For assessment of cell metabolic activity, MTT assay technique was used. Thereafter, a 20 μL of methyl thiazolyl tetrazolium (5 mg/mL, Sigma) was added to the plates. After removing dimethyl sulfoxide from the growth medium (100 μL), it was used for dissolving MTT crystal. The percentage of cell growth was calculated based on the optical densities for transfection (ODT) and the optical densities of control cells (ODC). The following formula was used for calculation: %control = ODT/ODC × 100. Also, half maximal inhibitory concentration (IC_50_) of each compound was reported from this assay (28). 

## Result and Discussion

*Chemical compounds identification*

By using step fractionation, two pale yellowish amorphous powders were isolated from *A. gigantea *aerial parts extract. Molecular weights of these compounds (compound **1 **and compound **2) **according to the exact mass analysis were determined 300.26, and 344.31 g/mol, respectively. Based on primary prediction from the ^1^H and ^13^C NMR (Table 1) data, it was estimated that these compounds might have flavonoid backbone. For compound **1**, the ^1^H NMR spectrum showed the presence of four substitutions on A and C ring of flavonoid core structure. Based on the ^13^C NMR data, due to the presence of five aromatic protons at δ 6.20 (H-6, 1H, d, J = 2 Hz), 6.62 (H-8, 1H, s), 7.48 (H-2’, 1H, d, J = 2 Hz), 6.94 (H-5’, 1H, d, J = 8.5 Hz), 7.52 (H-6’, 1H, dd, J = 8.5, 2 Hz), protons pic at δ 6.46 (H-3, 1H, d, J = 2 Hz) and singlet in δ 3.95, a mono methoxylated flavonoid structure was considered for compound **1**. The analysis of HMBC data of this compound showed the following correlations: (*a*) H-8 with C-6, C-7, C-9, C-10 in A ring, (*b*) H-3 with C-2, C-4, (*c*) H-2’ with C-3’, C-4’, C-2, and C-6’ in B ring, (*d*) H-5’ with C- 3’, C-4’, and C-1’ in B ring, and © C-5 of methoxyl group with H-5 in A ring. In addition to the pervious NMR informations, NOSEY and TOCSY data were reveled following correlation in compound 1 chemical structure: (from NOSEY spectrum) (a) H-6 with H-8, and vice versa, H-8 with H-6, (b) H-5’ with H-6’ and H-2’, (c) H-2’ with H-5’. (From TOCSY spectrum) (a) H-3 with H-2’, (b) H-6’ with H-3, and H-5’ (Table 1). 

In addition to these finding, by using the correlation between various carbons and protons in structure with TOCSY, COSY, DEPT, HSQC, and NOSEY techniques compound **1** structure was clarified as luteolin 5-methyl ether (supporting information was uploaded as a supplementary file) (29). 

For compound **2**, the ^1^H and ^13^C NMR showed similar pattern like the compound **1 (**Table 1**)**. Some important chemical shifts suggesting a flavone backbone are δ 154.4, 133.7, 95, 117.3, 122.4, and 152.8 ppm in ^13^C NMR. The proton chemical shifts are 6.67 (H-3, 1H, s), 6.58 (H-8, 1H, s), 7.49 (H-2’, 1H, d, J = 2 Hz), 7.11 (H-5’, 1H, d, J = 8.0 Hz), and 7.63 (H-6’, 1H, dd, J = 8.5, 2 Hz) in ^1^H NMR. Three singlets at δ 3.92, 3.91, and 3.87 represents the methoxy group in the structure. In addition to 1D NMR data, 2D NMR data confirmed the predicted three methoxylated flavone structure for compound **2**. The analysis of HMBC data of compound **2** showed the following correlations: (*a*) H-8 with C-10, C-6, C-9, C-7, C-4 in A ring, (*b*) H-3 with C-1’, C-2, C-10, C-4, (*c*) H-2’ with C-3’, C-4’, C-2, and C-6’ in B ring, (*d*) H-5’ with C- 3’, C-4’, and C-6’ in B ring, © H-6, H-7, and H-3’ of methoxyl groups with C-6, C-7, and C-2, respectively, in A ring (Table 1). For compound **2, **NOSEY and TOCSY spectrum showed very similar pattern to the compound **1. **Following correlations were identified for this compound: (from NOSEY spectrum) (*a*) H-3 with H-6’, (*b*) H-2’ with H-6’, (*c*) H-5’ with H-6’, and (*d*) H-6’ with H-2’, and H-5’. (From TOCSY spectrum) (*a*) H-2’ with H-5’, and H-6’ (*b*) H-6’ with H-2’, (*c*) H-5’ with H-2’. Based on these information in addition to the TOCSY, COSY, HSQC, and NOSEY data (Table 1 and the supporting information uploaded as a supplementary file compound 2 was identified as cirsilineol (30-33)**.**

In addition to these flavonoids, chemical compositions of the volatile oils of *A. gigantea *aerial parts and seed were identified. According to the GC-MS data the main constitutions of these volatile oils belong to the simple phenolic category which include coniferyl alcohol (18.80%) and eugenol (12.19%) in aerial parts and seeds, respectively (Table 2). 

*Molecular modelling results*

**Figure 2:**
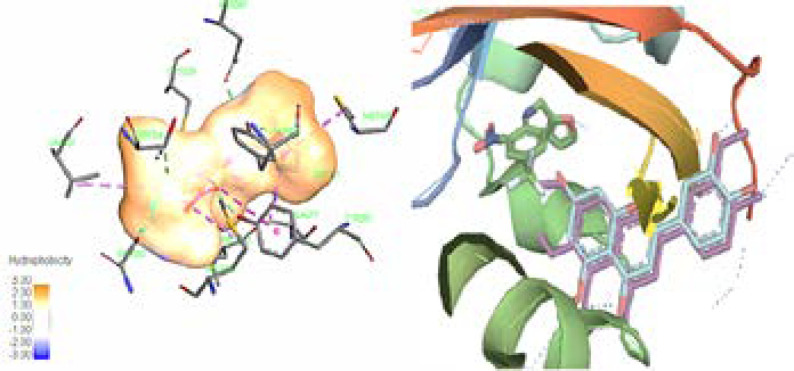
(Leaft) The ligand (020) in center of the binding pocket of 3H82, amino acids of the active box, and hydrophobicity map of binding pocket. (Right) The same view of binding pocket of 3H82 with ligand (020), luteolin 5-methyl ether, and cirsilineol. Chromen-4-one and C-ring of flavones clearly surrounded by the hydrophobic environment. Dotted line (dark blue) in B-ring showed potential of this part of flavones structures for hydrophilic interaction. All panels are shown in the same orientation. Active site analysis was carried out using Pymol & Discovery studio visualizer software

In this study, we calculated the free binding energy of luteolin 5-methyl ether (***1***) and cirsilineol (***2***) to four Homo sapiens AhR which are listed in Table 3. The better sub form of enzyme was chosen based on the matching of our compounds with active box for future analysis. Computer aided analysis indicated that besides the importance of the His248 and Tyr307 amino residues of 3H82 active box for creating hydrogen bond for its ligand (020), hydrophobic environment which include Phe244, Phe254, Phe280, Tyr307, Met309, and Leu319 amino acids side chains play notable role in interaction between ligand and enzyme (34). It is evident that like other specific ligands of this enzyme, luteolin 5-methyl ether (***1***) and cirsilineol (***2***) have also shown direct hydrophobic interactions with this hydrophobic box residues, which mostly involve the “A & C-ring” of these flavonoids (35). Although Tyr281 could show the π-π interaction with aromatic “C-ring” of these compounds, it could also act as important residues in active box especially in the presence of “C-ring hydroxyl groups” of the flavonoids (Figure 2) (36). Our finding showed that in this series of enzyme, the backbone of chromen-4-one is probably surrounded by the hydrophobic environment. In contrast, “B-ring” of the flavonoids could also play an important role in hydrophilic interaction especially by His248 and Tyr307 in active box of 3H82 (37). Based on previous finding, the flavonoids or their derivatives with the 7- and 8-carbons in C-ring, 1-pyran and a B-ring, have shown affinity for the hydrophobic pocket of AhR which confirmed our *in-silico* finding (Figure 2) (36). 

Working on the cytotoxicity profiles characterization of the methoxylated and other lipophilic flavonoids on carcinogenic cells led to the identification of some structure-activity relationships (SAR) of these compounds (34, 35). Based on SAR studies, hydroxyl substituents at C-3′ and C-5 and methoxyl groups at C-4′ have the most effect on this potency in flavones (38). Recent laboratory studies have demonstrated the important role of AhR/CYP systems in cancer initiation, resistance, and progression (37, 39). As AhR/CYP chemical inhibitors, these classes of compounds have shown high activity in some *in-vitro* assay(40). AhR receptors are ligand-activated factors which regulate various cell functions such as differentiation, proliferation as well as reproduction(41). AhR activation by different ligand, such as benzo[a]pyrene (BaP) or 7, 12-dimethybenz[a]anthracene (DMBA), could initiate this activated transcriptional factor for regulating the number of genes such as the cytochrome P450 1B1 (CYP1B1) and CYP1A1(42). Induction of these cytochromes is a well-known mechanism for the development of epoxide and diol-epoxide carcinogenic intermediates which finally form DNA adducts and triggered tumor initiation (43). Therefore, inactivation of AhR/ cytochrome system using the chemical inhibitor could be considered as a valuable point for cancer chemoprevention and chemotherapy development (31, 44).

In summary, molecular models showed that AhR-flavones could be considered as the suitable target for more studies for finding lead structures with good anticancer potential. Based on molecular molding, it could be predicted that adding the hydrophobic group at B-ring of flavones structures creates more tendency for the AhR active box. In contrast, adding the polar group to B-ring of these structures often caused decrease in the tendency for the AhR active box. Methylation of the 7-OH, and 3’ -OH in flavone backbone (in cirsilineol) possibility amplified interaction between compound and AhR active box compared to the less methoxylated flavone, luteolin 5-methyl ether. However, according to the pervious investigation methylation of 6-OH in A ring of flavone backbone could decrease tendency for active box but in the present investigation this structural change in flavone backbone didn’t attenuate the interaction with active box.

Overall, according to the *in silico* result in the present study, methoxylated flavones isolated from *A. gigantea* are able to interact with AhR and probably play agonistic, antagonistic or even neutral effect for cancer cells (Table 3 & Figure 2).

*MTT assay results*

The widespread of the flavonoids class of chemical compounds in plants and their useful potential in cancer prevention have attracted huge attention to the screening of their anticancer potential. Although many of these class of compounds don’t have high toxicity against normal cells, numerous investigations showed their cytotoxic potential against various carcinogenic cell lines (33). These anticancer potentials of the various flavonoids have attracted huge attention for finding natural or synthetic flavonoids related compounds as lead anticancer compounds. 

**Figure 3:**
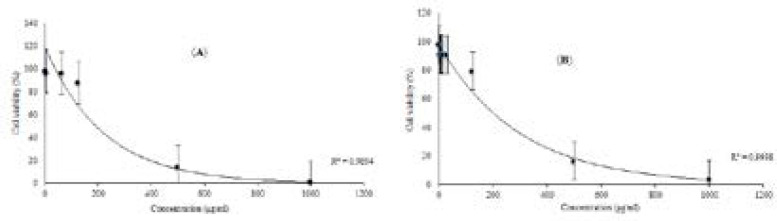
Effects of luteolin 5-methyl ether (***a***) and cirsilineol (***b***) on the viability of 4T1 cell for 24 h, respectively. 4T1 cell was treated with various indicated concentrations of two compounds, separately. MTT assay was used for cell viability determination, this results were expressed as mean±S.D. of three separate experiments. Luteolin 5-methyl ether and cirsilineol were shown very close activity, the IC_50_ values on cytotoxicity were 428.24 ±3.21 and 412.7±3.02 μg/mL. Significant differences are indicated by **p < 0.01

Therefore, in the following *in-silico* assay, cytotoxic potential of luteolin 5-methyl ether and cirsilineol was tested against the 4T1 breast carcinoma cell line which is well known for AhR oncogenic functions in carcinogenicity(45). The cytotoxicity and antiproliferative activity of luteolin 5-methyl ether (1) and cirsilineol (2) against 4T1 cell are shown in Figure 3. At concentration of 500 μg/mL, both compounds showed cytotoxic effect against 4T1 cell. The IC_50_ values on cytotoxicity were 428.24 ±3.21 and 412.7±3.02 μg/mL for luteolin 5-methyl ether and cirsilineol, respectively. Thus, it can be concluded that these flavonoids did not exhibit significant cytotoxicity against 4T1 breast carcinoma cell line.

In many cases, results from *in-silico* experiments cannot be directly extrapolated to *in-vitro* effects. This opposed *in-vitro* results could be explained by previous investigation outputs, many of the flavone based structures such as 6, 2’, 4’-trimethoxyflavone could exhibit significant antagonistic effect on AhR activity but other related compounds of this class such as β-naphthoflavone, quercetin, kaempferol, diosmin, and diosmetin exhibited agonistic activity for AhR (46, 47).

**Table 1:** ^1^H NMR, ^13^ C NMR and 2D NMR Correlations Data of luteolin 5-methyl ether (***1***) and cirsilineol (***2***)

***Position***	^1^ **H NMR**		^13^ C NMR**		**TCOSY **		**HMBC**		**COSY**		**NOESY**	
	**δ **_H_ ** mult. (J in Hz)**						**(H→C)**		**(H→H)**			
	**Com. 1**	**Com. 2**	**Com. 1**	**Com. 2**	**Com. 1**	**Com. 2**	**Com. 1**	**Com. 2**	**Com. 1**	**Com. 2**	**Com. 1**	**Com. 2**
**1**	-	-	-	-	-	-	-	-	-	-	-	-
**2**	-	-	165.5	-	-	-	-	-	-	-	-	-
**3**	6.46, d (2)	6.67, s	105.8	-	-	-	2, 4	1’, 2, 4, 10	-	-	-	6’
**4**	-	-	183.4	184.9	-	-	-	-	-	-	-	-
**5**	-	-	159.5	-	-	-	-	-	-	-	-	-
**6**	6.20, d (2)	-	100.0	133.7	8	-	-	-	-	-	-	-
**7**	-	-	166.1	154.4	-	-	-	-	-	-	-	-
**8**	6.62,s	6.58, s	95.1	95	6	-	7, 9, 10, 6	10, 6, 9, 7, 4	-	-	-	-
**9**	-	-	158.3	159.4	-	-	-	-	-	-	-	-
**10**	-	-	103.7	105.7	-	-	-	-	-	-	-	-
**1’**	-	-	124.1	124.4	-	-	-	-	-	-	-	-
**2’**	7.48, d (2)	7.49, d (2)	110.3	111.6	5’	6’, 5’	3’, 4’, 2, 6’	6’, 3’, 4’, 2	-	6’	3	6’
**3’**	-	-	149.7	151.6	-	-	-	-	-	-	-	-
**4’**	-	-	152.8	153.8	-	-	-	-	-	-	-	-
**5’**	6.94, d (8.5)	7.11, d (8)	117.3	113.7	6’, 2’	2’	3’, 4’, 1’	6’, 3’, 4’	6’	6’	-	6’
**6’**	7.52, dd (8.5,2)	7.63, dd (8, 2)	122.4	121.2	5’	2’	4’	2’, 4’	5’	5’, 2’	3, 5’	5’, 2’
**(in 5) OCH**_3_	3.95, s	-	56.3	-	-	-	5	-	-	-	-	-
**(in 6) OCH3**	-	3.92, s	-	56.1	-	-	-	7	-	-	-	-
**(in 7) OCH3**	-	3.91, s	-	56.3	-	-	-	6	-	-	-	-
**(in 3’) OCH3**		3.87 s	-	61.2	-	-	-	2	-	-	-	-

* All ^1^H assignments are based on 2D NMR (COSY, HSQC and HMBC) experiments.

**^13^C assignments are based on HSQC and HMBC.

**Table 2.** Chemical compositions of the *A. gigantea *aerial parts and seed volatile oils

	**Compound name**	**Aerial parts**	**Seed **		
					
		HD (%) ^c^	HD (%) ^c^	RI ^a^	RI ^b^
					
1	2-ethyl-1-hexanol	-	1.1	1032	1028
2	1-methyl-2-pyrolidinone	3.7	0.8	1034	1030
3	2-ethenyl-1,3,3-trimethyl-cyclohexane	0.8	-	-	1132
4	2, 3-dihydro-Benzofuran	5.1	-	1226	1221
5	Ascaridole epoxide	0.8	3.2	-	1245
6	Thymol	-	8.1	1292	1289
7	Eugenol	-	15.1	1356	1354
8	α- Copaene	-	0.5	1377	1375
9	Chavibetol	7.1	-	1392	1387
10	6-methoxy-2-methyl-tetracyclo[5.3.1.0(2,6).0(8.11)undecan-4-ol	-	0.9	-	1389
11	β-Cubebene	-	0.6	1390	1392
12	Vanillin	8.1	-	1405	1400
13	β- Caryophyllene	-	5.4	1420	1421
14	1,4-Dimethoxy-2,3-dimethylbenzene	3.1	-	-	1442
15	γ-muurolene	-	0.6	1477	1479
16	4-(5,5-dimethyl-1-oxaspiro[2.5]oct-4-yl)3-buten-2-one	0.5	-	1493	1489
17	*Cis*-β-Guaiene	-	0.7	1491	1490
18	β-Ionone	0.5	-	1503	1500
19	γ-Cadinene	-	4.6	1514	1518
20	4a-acetoxy-5,5,8a-trimethyl-octahydrobenzo[b]pyran	-	0.5	-	1521
21	3-(2,2,3,3-tetramethylcyclopropylidenmethylidene)-4-methyl-hexanoic acid	-	0.6	-	1529
22	Dihydroactinidiolide	6.2	3.2	1532	1534
23	*Trans*-4-Propenylsyringol	0.4	-	-	1536
24	Spatulenol	-	0.6	1576	1578
25	4-(Acetyloxy)-3-methoxy-methylester benzoic acid	0.7	-	-	1660
26	Coniferyl alcohol	18.8	-	-	1682
27	Acorenone	0.5	-	1693	1690
28	4-hydroxy-3,5,5-trimethyl-4-(3-oxo-1-butenyl)-2-cyclohexen-1-one	0.5	-	-	1787
29	Dehydrovomifoliol	4.7	-	1796	1793
30	Decahydro-1,5,5,8a-tetramethyl-[1S-(1α,3β,3aβ,4α,8aα)]-1,4-methanoazulen-3-ol	-	2.1	-	1827
31	2-(2,2,6-trimethyl-7-oxa-bicyclo[4.1.0]hept-1-yl)-propenyl ester acetic acid	15.1	-	-	1832
32	Methyl linoleate	-	6.8	2096	2092
33	Octadecanoic acid	-	10.4	2158	2154
34	3',4',5'-O-trimethyltricetin	1.5	-	-	2521
35	α-tocopherol	3.1	1.1	3111	3108
36	Stigmasterol	-	0.8	3170	3165
37	β-Sitosterol	-	0.5	3203	3200
					
					
		**81.2**	**69.8**		

^a^ Retention indices given in literature (NIST on non-polar HP-5 or DB-5 capillary columns).

^b^ Retention indices with respect to C5–C28 n-alkanes calculated on non-polar DB-5 capillary column.

^c^ Percentage calculated by GC-FID on non-polar DB-5 capillary column.

**Table 3:** The best free binding energies calculated values (EFEB) and root-mean-square deviation of atomic positions (rmsd l.b. & u.b.) of PDB ligands, luteolin 5-methyl (***1***) ether and cirsilineol (***2***) for four various *Homo sapiens* aryl hydrocarbon receptor

***AhR receptor***	***PDB ligand***			***luteolin 5-methyl***			***cirsilineol***		
	EFEB (kcal/mol)	rmsd l.b.	rmsd u.b.	EFEB (kcal/mol)	rmsd l.b.	rmsd u.b.	EFEB (kcal/mol)	rmsd l.b.	rmsd u.b.
*5UFP*	-7.0	0.000	0.000	-6.3	0.880	2.030	-6.8	1.436	2.132
*5TBM*	-10.2	0.000	0.000	-6.3	0.988	2.082	-7.5	1.150	2.570
*4XT2*	-9.0	0.000	0.000	-6.7	1.018	2.112	-7.3	1.133	2.422
*3H82*	-7.1	0.000	0.000	-6.3	1.071	2.149	-7.1	1.854	1.871

## Conclusion

In the present study, two flavonoids were isolated from *A. gigantea* aerial parts. After structure elucidation with different NMR techniques they were identified as luteolin 5-methyl ether and cirsilineol. Since numerous investigations have shown cytotoxic potential against various carcinogenic cell lines, the cytotoxicity of these molecules were evaluated by *in-silico* and *in-vitro* studies. According to the *in-silico* study, methoxylated flavones isolated from *A. gigantea* were able to interact with AhR, appropriately. But, the *in-vitro *assays showed low cytotoxicity for luteolin 5-methyl ether and cirsilineol against 4T1 cell. It can be concluded that high affinity of flavones structures to the aryl hydrocarbon receptor does not guarantee more cytotoxic potential. However, the *in-silico* methods could be an open valuable line for purposeful assays on natural compounds. 

## Supporting Online Material

Supplementary Material
