# Supplementary material for: Phytochemical Investigation on Volatile Compositions and Methoxylated Flavonoids of Agrostis gigantea Roth
Source: Iran J Pharm Res. 2020 Spring;19(2):360–70. doi: 10.22037/ijpr.2019.15209.12935 (PMC7667570; doi:10.22037/ijpr.2019.15209.12935)
Supplement: Supplementary Material [file ijpr-19-360.s001.pdf]

Supplementary Materials for

Phytochemical investigation on volatile compositions and methoxylated flavonoids of *Agrostis gigantea* Roth

Mahmoud Rafieian-kopaei, Azadeh Hamedi, Ebrahim Soleiman Dehkordi, Arsalan Pasdaran and Aradalan Pasdaran*

*To whom correspondence should be addressed

pasdaran@sums.ac.ir

Volume 19, Issue 2 (Spring 2020)

This PDF file include

Figures s1to s13

1D, 2D NMR data of luteolin 5-methyl ether.

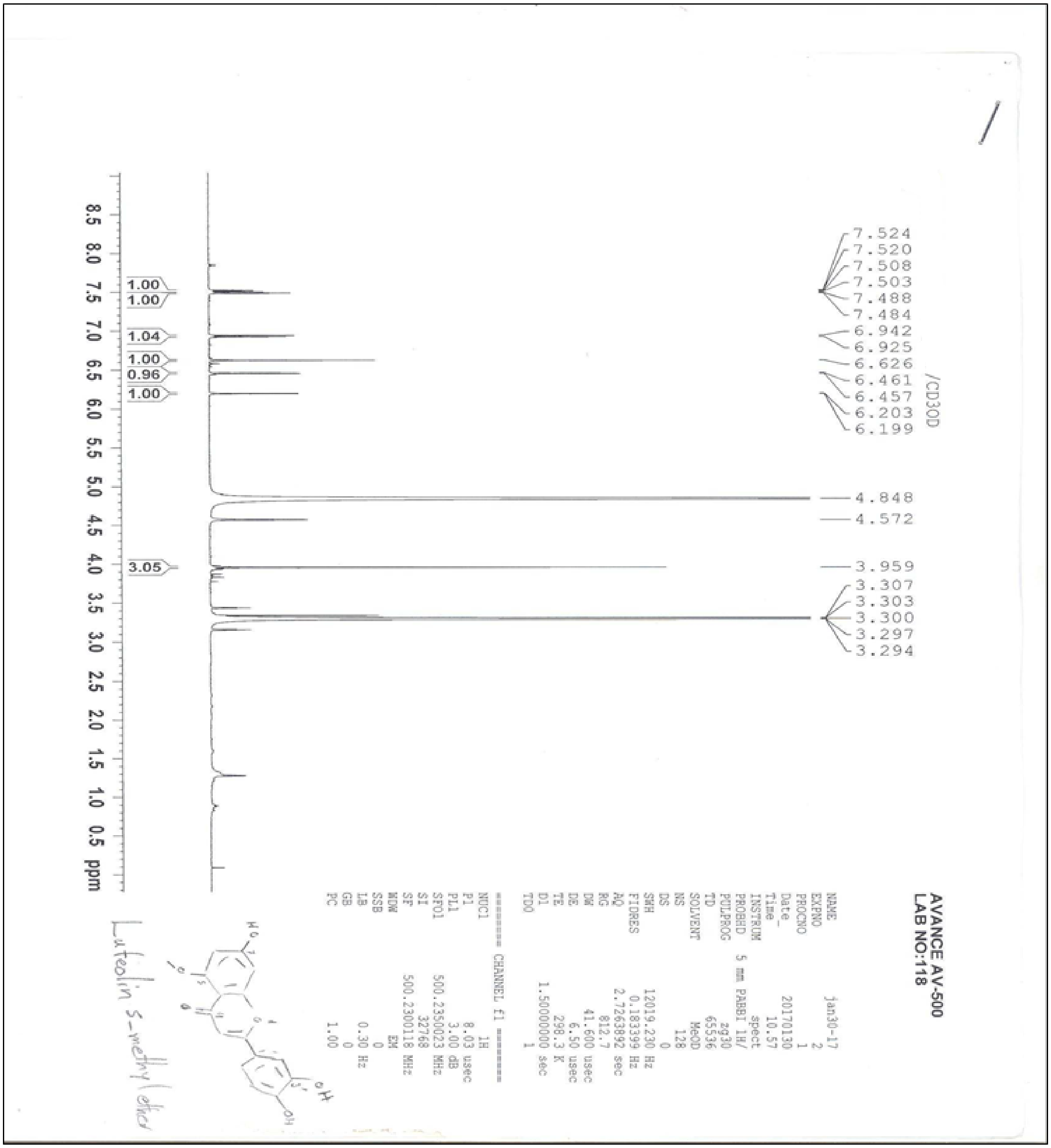

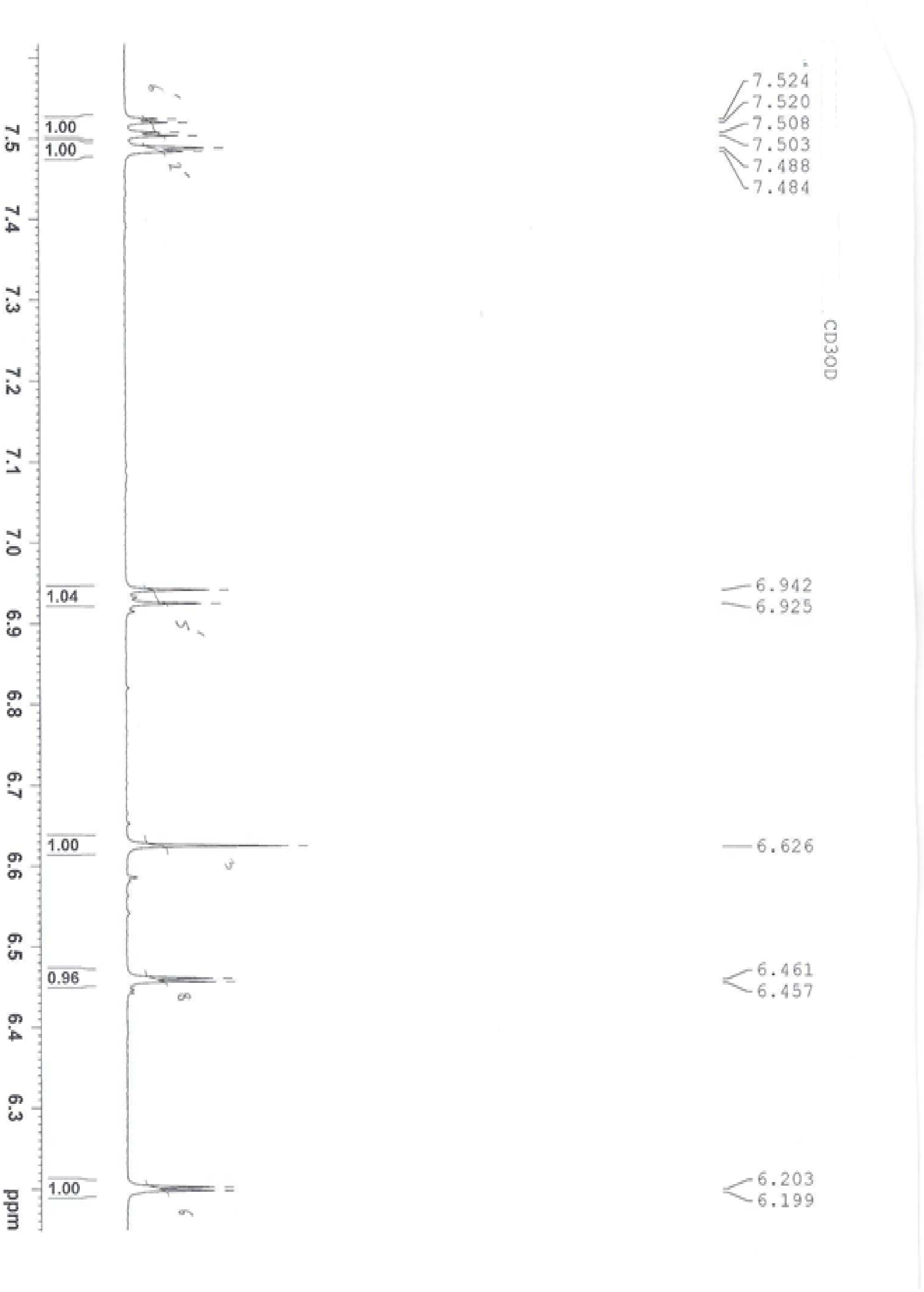
7.524
7.520
7.508
7.503
7.488
7.484
CD3OD
6.942
6.925
6.626
6.461
6.457
6.203
6.199
1.00
1.00
1.04
1.00
0.96
1.00
7.5
7.4
7.3
7.2
7.1
7.0
6.9
6.8
6.7
6.6
6.5
6.4
6.3
ppm

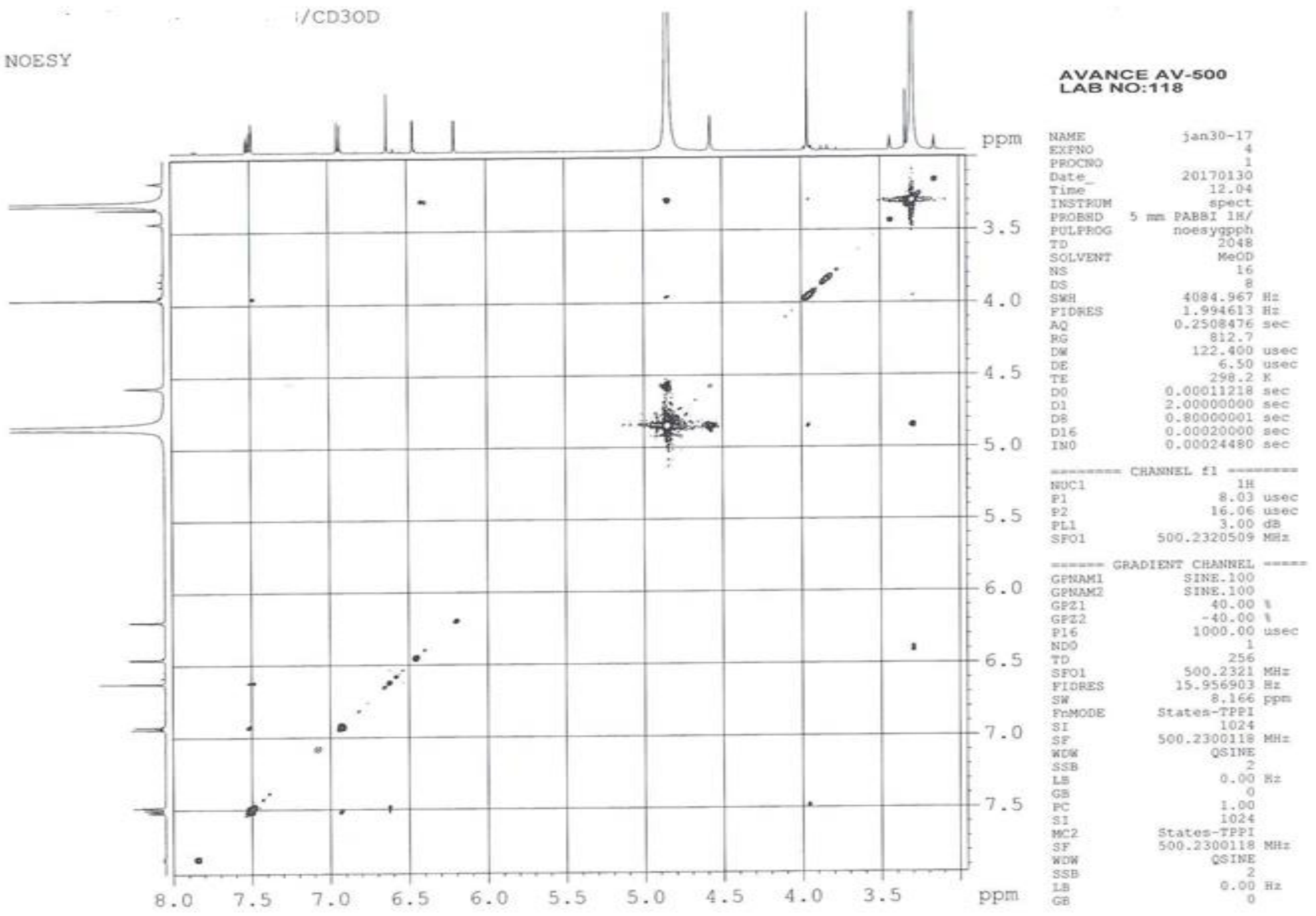
/CD3OD
NOESY
AVANCE AV-500
LAB NO:118
ppm
3.5
4.0
4.5
5.0
5.5
6.0
6.5
7.0
7.5
8.0
7.5
7.0
6.5
6.0
5.5
5.0
4.5
4.0
3.5
ppm
NAME jan30-17
EXPNO 4
PROCNO 1
Date_ 20170130
Time 12.04
INSTRUM spect
PROBHD 5 mm PABBI 1H/
PULPROG noesygpph
TD 2048
SOLVENT MeOD
NS 16
DS 8
SWH 4084.967 Hz
FIDRES 1.994613 Hz
AQ 0.2508476 sec
RG 812.7
DW 122.400 usec
DE 6.50 usec
TE 298.2 K
D0 0.00011218 sec
D1 2.00000000 sec
D8 0.80000001 sec
D16 0.00020000 sec
IN0 0.00024480 sec
======== CHANNEL f1 ========
NUC1 1H
P1 8.03 usec
P2 16.06 usec
PL1 3.00 dB
SFO1 500.2320509 MHz
====== GRADIENT CHANNEL =====
GPNAM1 SINE.100
GPNAM2 SINE.100
GPZ1 40.00 %
GPZ2 -40.00 %
P16 1000.00 usec
ND0 1
TD 256
SFO1 500.2321 MHz
FIDRES 15.956903 Hz
SW 8.166 ppm
FnMODE States-TPPI
SI 1024
SF 500.2300118 MHz
WDW QSINE
SSB 2
LB 0.00 Hz
GB 0
PC 1.00
SI 1024
MC2 States-TPPI
SF 500.2300118 MHz
WDW QSINE
SSB 2
LB 0.00 Hz
GB 0

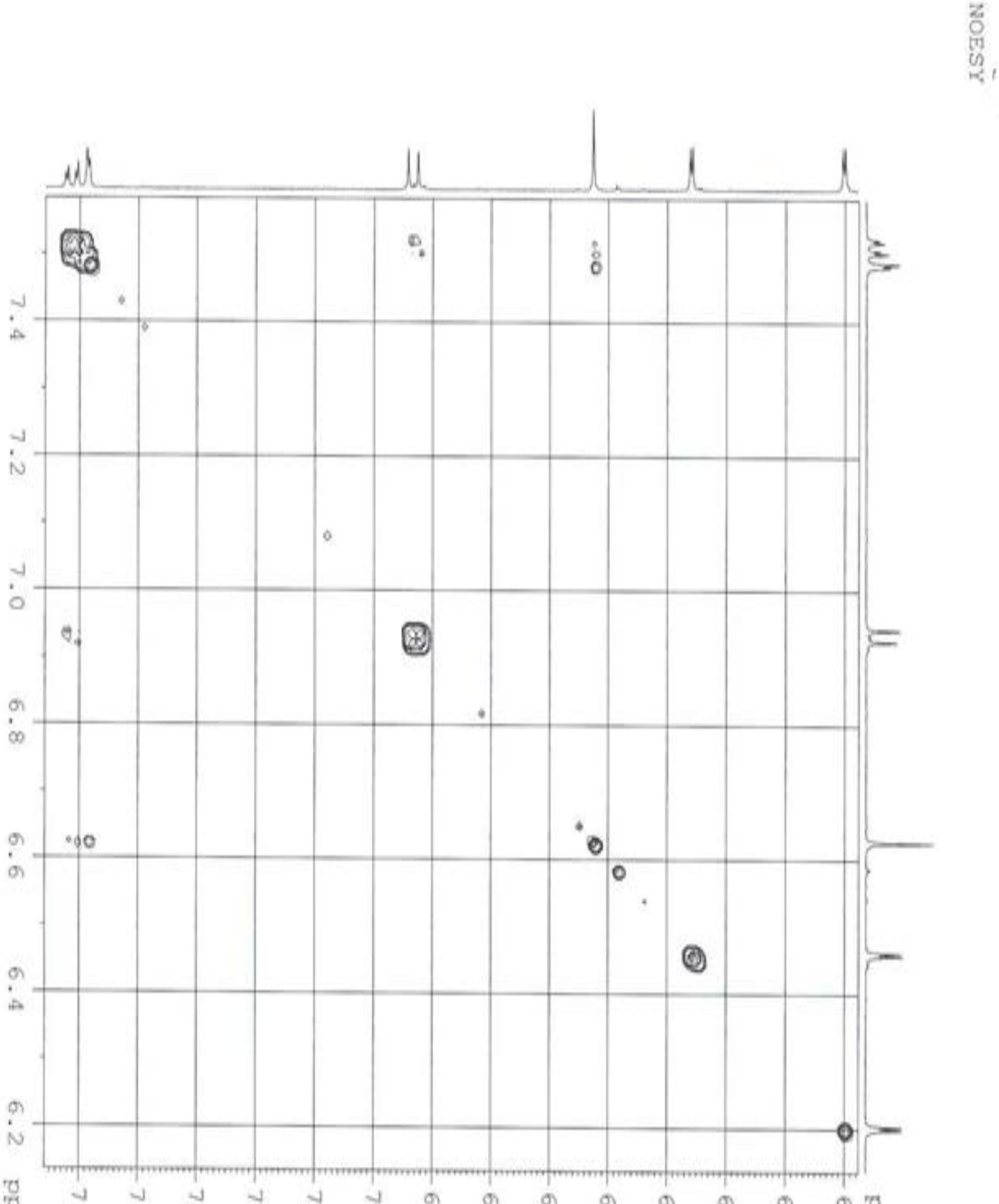
NOESY
/CD3OD
ppm
ppm

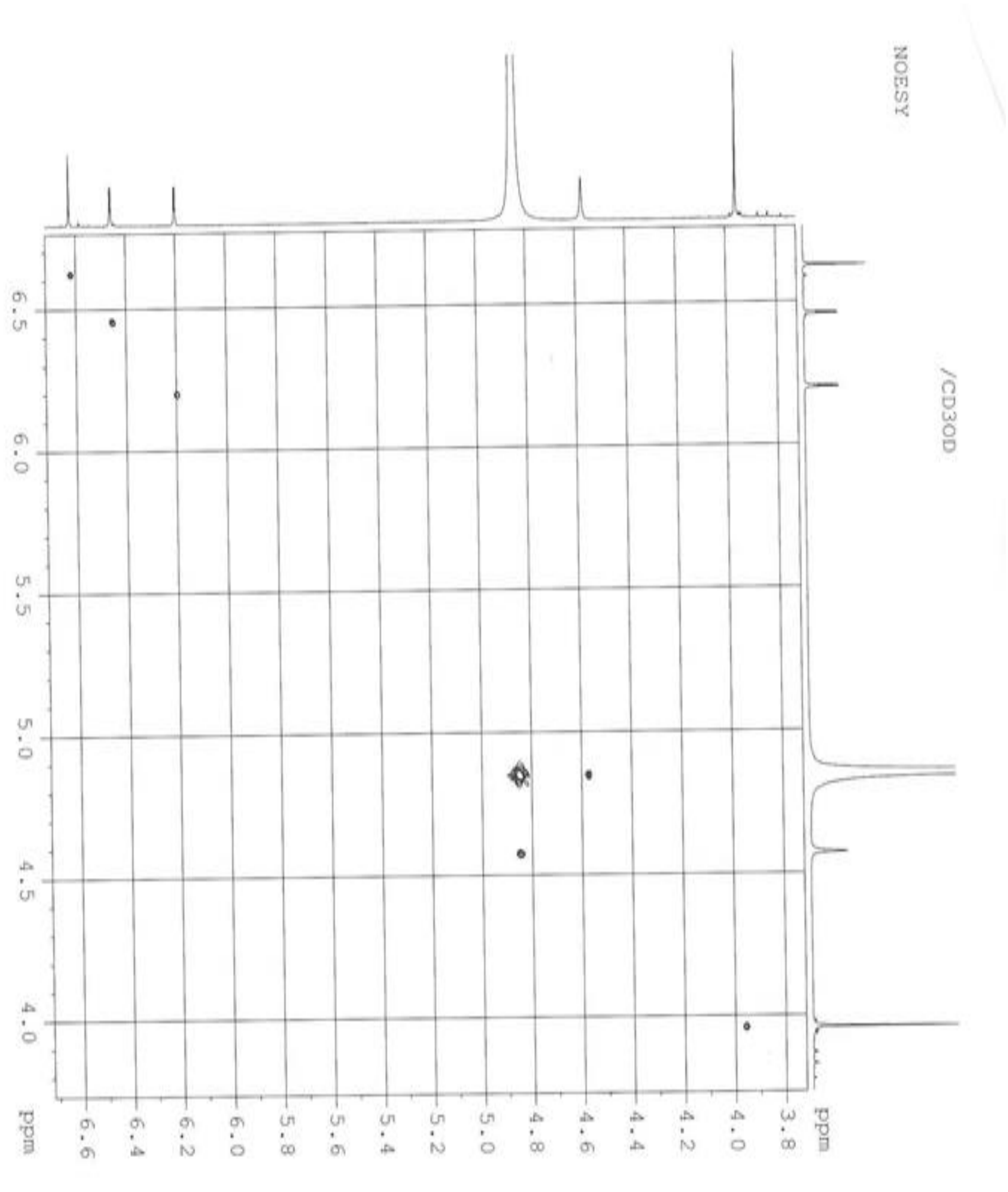
NOESY
/CD3OD
ppm
ppm

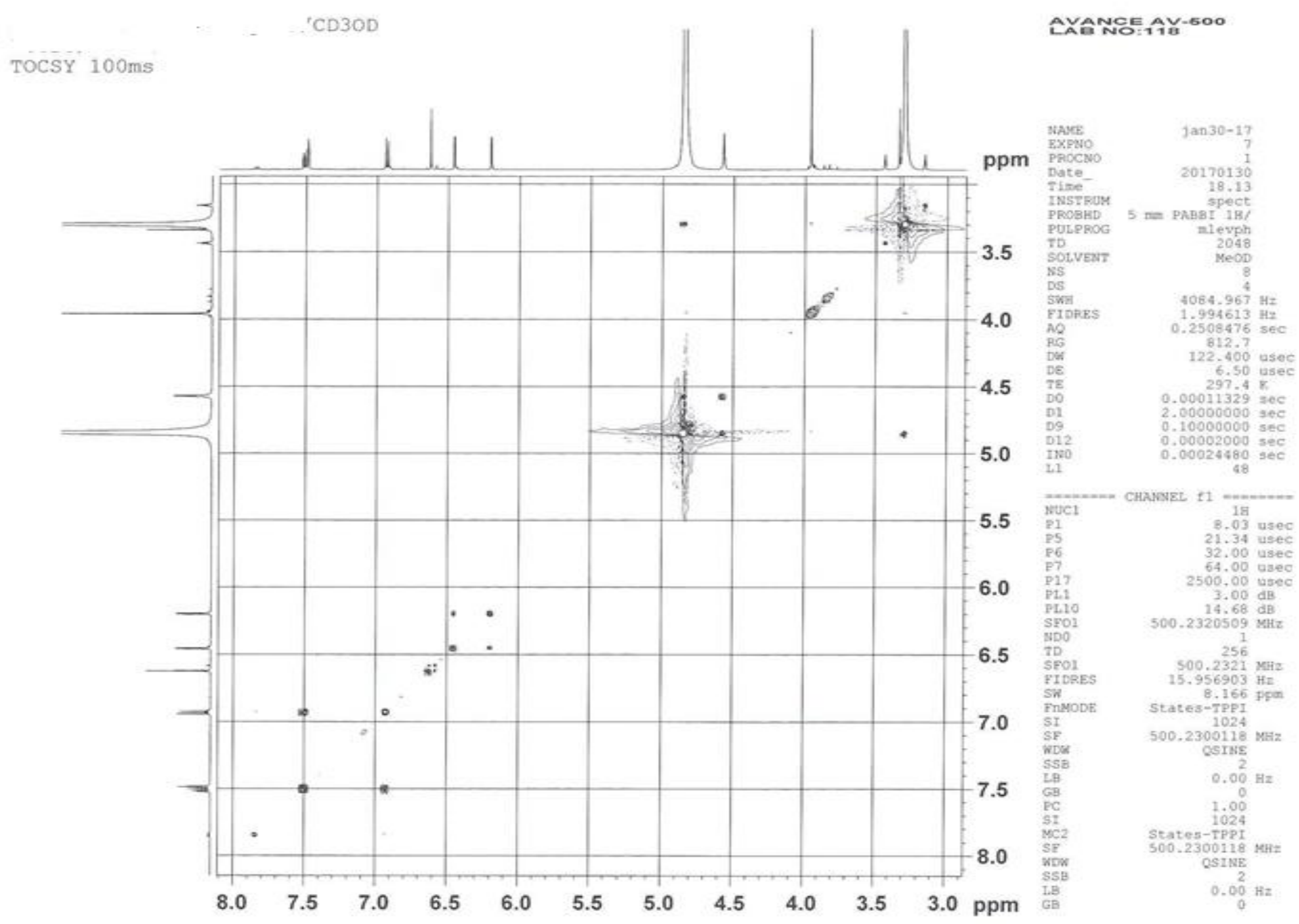
'CD3OD
TOCSY 100ms
AVANCE AV-500
LAB NO:118
NAME jan30-17
EXPNO 7
PROCNO 1
Date_ 20170130
Time 18.13
INSTRUM spect
PROBHD 5 mm PABBI 1H/
PULPROG mlevph
TD 2048
SOLVENT MeOD
NS 8
DS 4
SWH 4084.967 Hz
FIDRES 1.994613 Hz
AQ 0.2508476 sec
RG 812.7
DW 122.400 usec
DE 6.50 usec
TE 297.4 K
D0 0.00011329 sec
D1 2.00000000 sec
D9 0.10000000 sec
D12 0.00002000 sec
IN0 0.00024480 sec
L1 48
======== CHANNEL f1 ========
NUC1 1H
P1 8.03 usec
P5 21.34 usec
P6 32.00 usec
P7 64.00 usec
P17 2500.00 usec
PL1 3.00 dB
PL10 14.68 dB
SFO1 500.2320509 MHz
ND0 1
TD 256
SFO1 500.2321 MHz
FIDRES 15.956903 Hz
SW 8.166 ppm
FnMODE States-TPPI
SI 1024
SF 500.2300118 MHz
WDW QSINE
SSB 2
LB 0.00 Hz
GB 0
PC 1.00
SI 1024
MC2 States-TPPI
SF 500.2300118 MHz
WDW QSINE
SSB 2
LB 0.00 Hz
GB 0
ppm
3.5
4.0
4.5
5.0
5.5
6.0
6.5
7.0
7.5
8.0
8.0 7.5 7.0 6.5 6.0 5.5 5.0 4.5 4.0 3.5 3.0 ppm

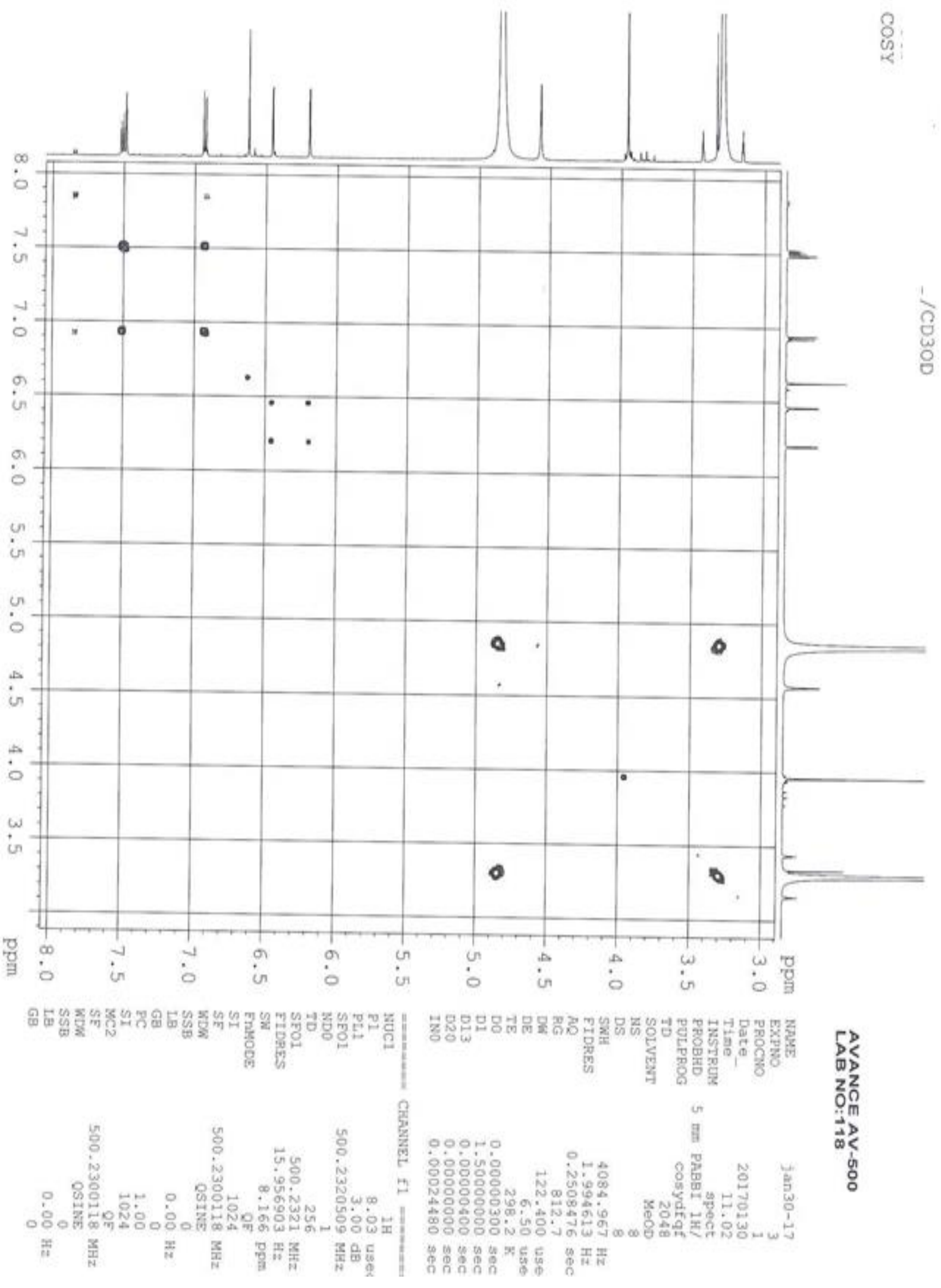
COSY
_/CD3OD
AVANCE AV-500
LAB NO:118
8.0
7.5
7.0
6.5
6.0
5.5
5.0
4.5
4.0
3.5
3.0
ppm
NAME jan30-17
EXPNO 3
PROCNO 1
Date_ 20170130
Time 11.02
INSTRUM spect
PROBHD 5 mm PABBI 1H/
PULPROG cosydfqf
TD 2048
SOLVENT MeOD
NS 8
DS 8
SWH 4084.967 Hz
FIDRES 1.994613 Hz
AQ 0.2508476 sec
RG 812.7
DW 122.400 use
DE 6.50 use
TE 298.2 K
D0 0.00000300 sec
D1 1.50000000 sec
D13 0.00000400 sec
D20 0.00000000 sec
IN0 0.00024480 sec
======== CHANNEL f1 ========
NUC1 1H
P1 8.03 use
PL1 3.00 dB
SFO1 500.2320509 MHz
ND0 1
TD 256
SFO1 500.2321 MHz
FIDRES 15.956903 Hz
SW 8.166 ppm
FnMODE QF
SI 1024
SF 500.2300118 MHz
WDW QSINE
SSB 0
LB 0.00 Hz
GB 0
PC 1.00
SI 1024
MC2 QF
SF 500.2300118 MHz
WDW QSINE
SSB 0
LB 0.00 Hz
GB 0

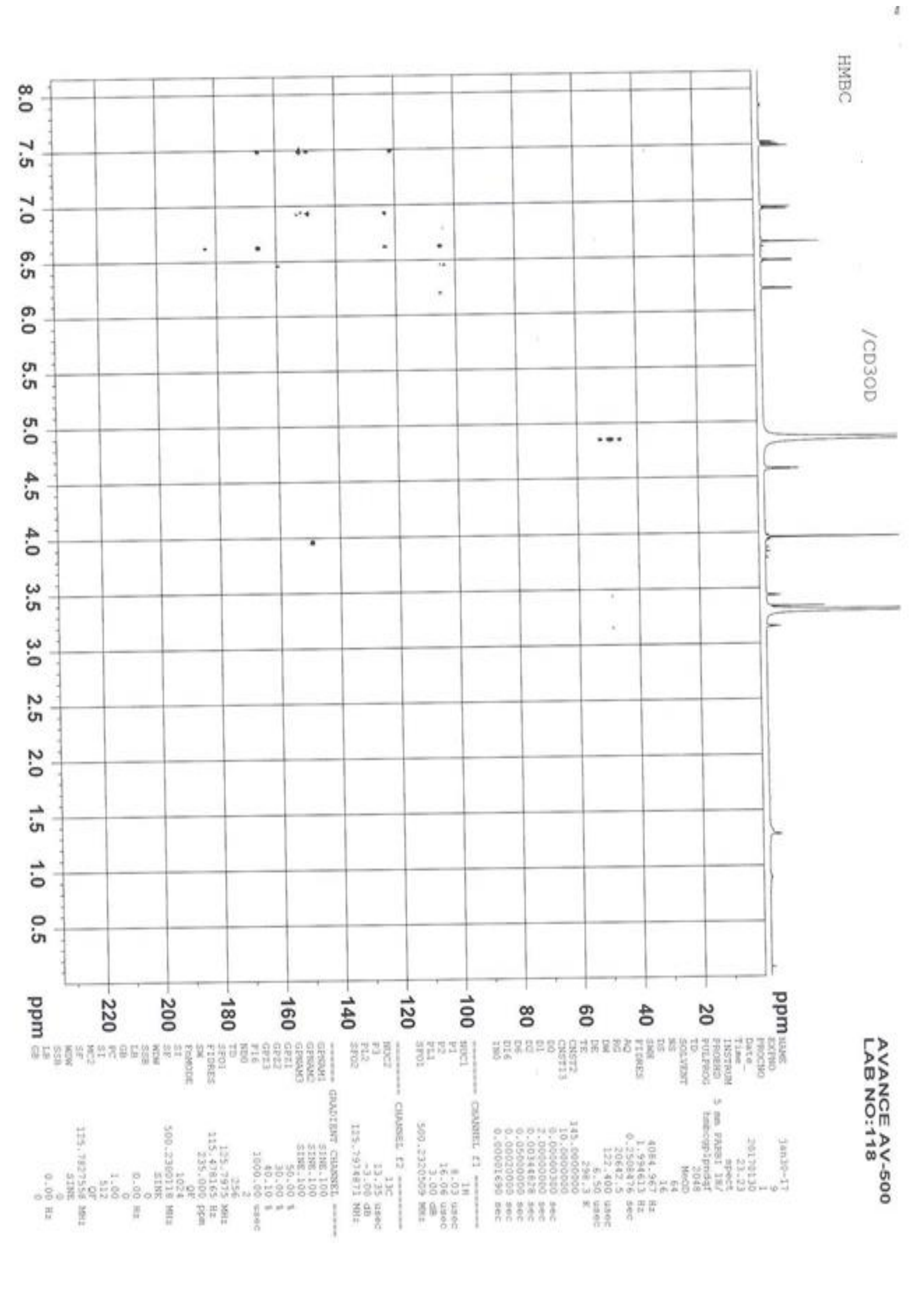
HMBC
/CD3OD
AVANCE AV-500
LAB NO:118

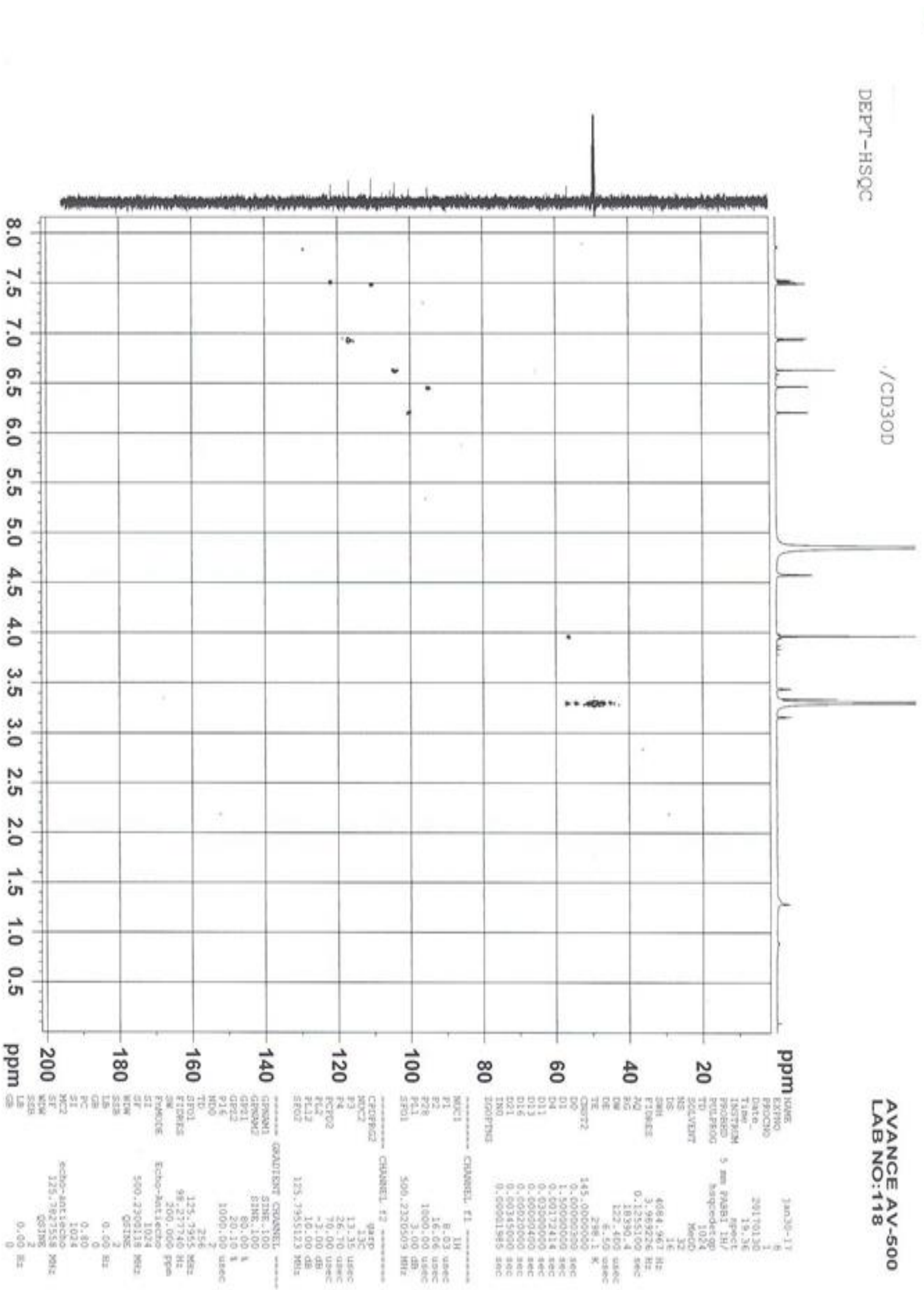
DEPT-HSQC
/CD3OD
AVANCE AV-500
LAB NO:118

1D, 2D NMR data of cirsilineol.

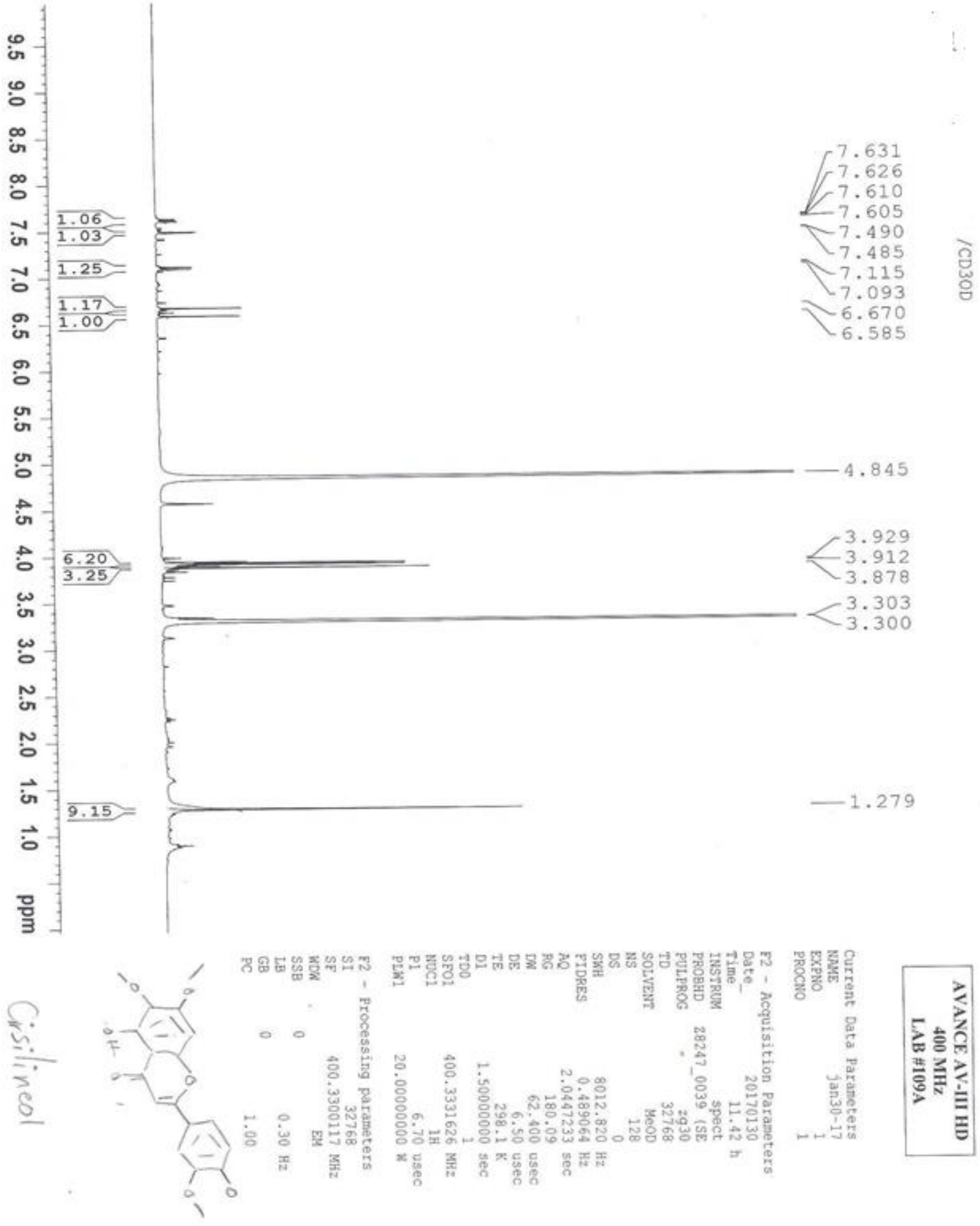

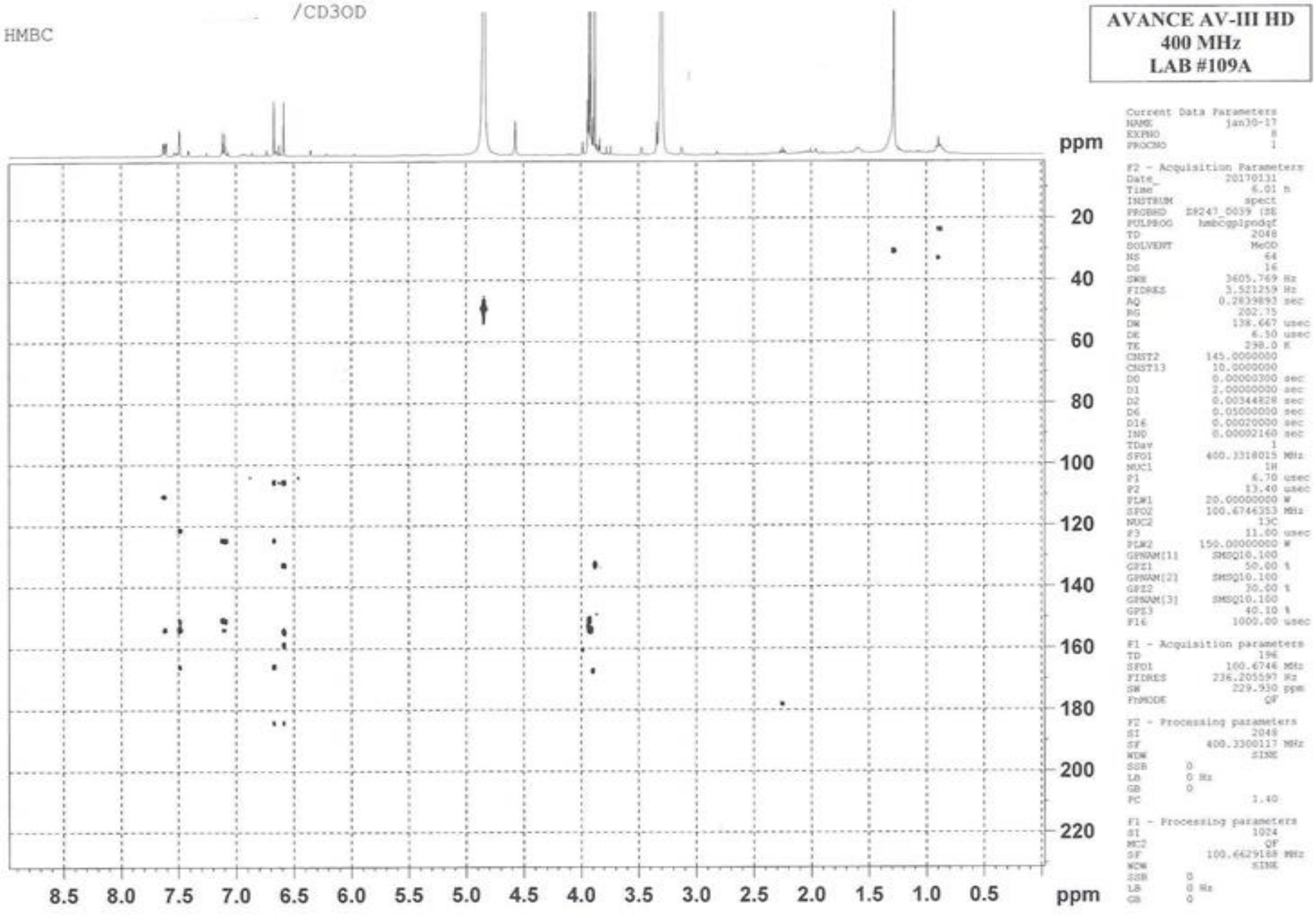
HMBC
/CD3OD
AVANCE AV-III HD
400 MHz
LAB #109A
Current Data Parameters
NAME jan30-17
EXPNO 8
PROCNO 1
F2 - Acquisition Parameters
Date_ 20170131
Time 6.01 h
INSTRUM spect
PROBHD Z8247_0039 (SE
PULPROG hmbcgplpndqf
TD 2048
SOLVENT MeOD
NS 64
DS 16
SWH 3605.769 Hz
FIDRES 3.521259 Hz
AQ 0.2839893 sec
RG 202.75
DW 138.667 usec
DE 6.50 usec
TE 298.0 K
CNST2 145.0000000
CNST13 10.0000000
D0 0.00000300 sec
D1 2.00000000 sec
D2 0.00344828 sec
D6 0.05000000 sec
D16 0.00020000 sec
IN0 0.00002160 sec
TDav 1
SFO1 400.3318015 MHz
NUC1 1H
P1 6.70 usec
P2 13.40 usec
PLW1 20.00000000 W
SFO2 100.6746353 MHz
NUC2 13C
P3 11.00 usec
PLW2 150.00000000 W
GPNAM[1] SMSQ10.100
GPZ1 50.00 %
GPNAM[2] SMSQ10.100
GPZ2 30.00 %
GPNAM[3] SMSQ10.100
GPZ3 40.10 %
P16 1000.00 usec
F1 - Acquisition parameters
TD 196
SFO1 100.6746 MHz
FIDRES 236.205597 Hz
SW 229.930 ppm
FnMODE QF
F2 - Processing parameters
SI 2048
SF 400.3300117 MHz
WDW SINE
SSB 0
LB 0 Hz
GB 0
PC 1.40
F1 - Processing parameters
SI 1024
MC2 QF
SF 100.6629188 MHz
WDW SINE
SSB 0
LB 0 Hz
GB 0
ppm
20
40
60
80
100
120
140
160
180
200
220
8.5 8.0 7.5 7.0 6.5 6.0 5.5 5.0 4.5 4.0 3.5 3.0 2.5 2.0 1.5 1.0 0.5 ppm

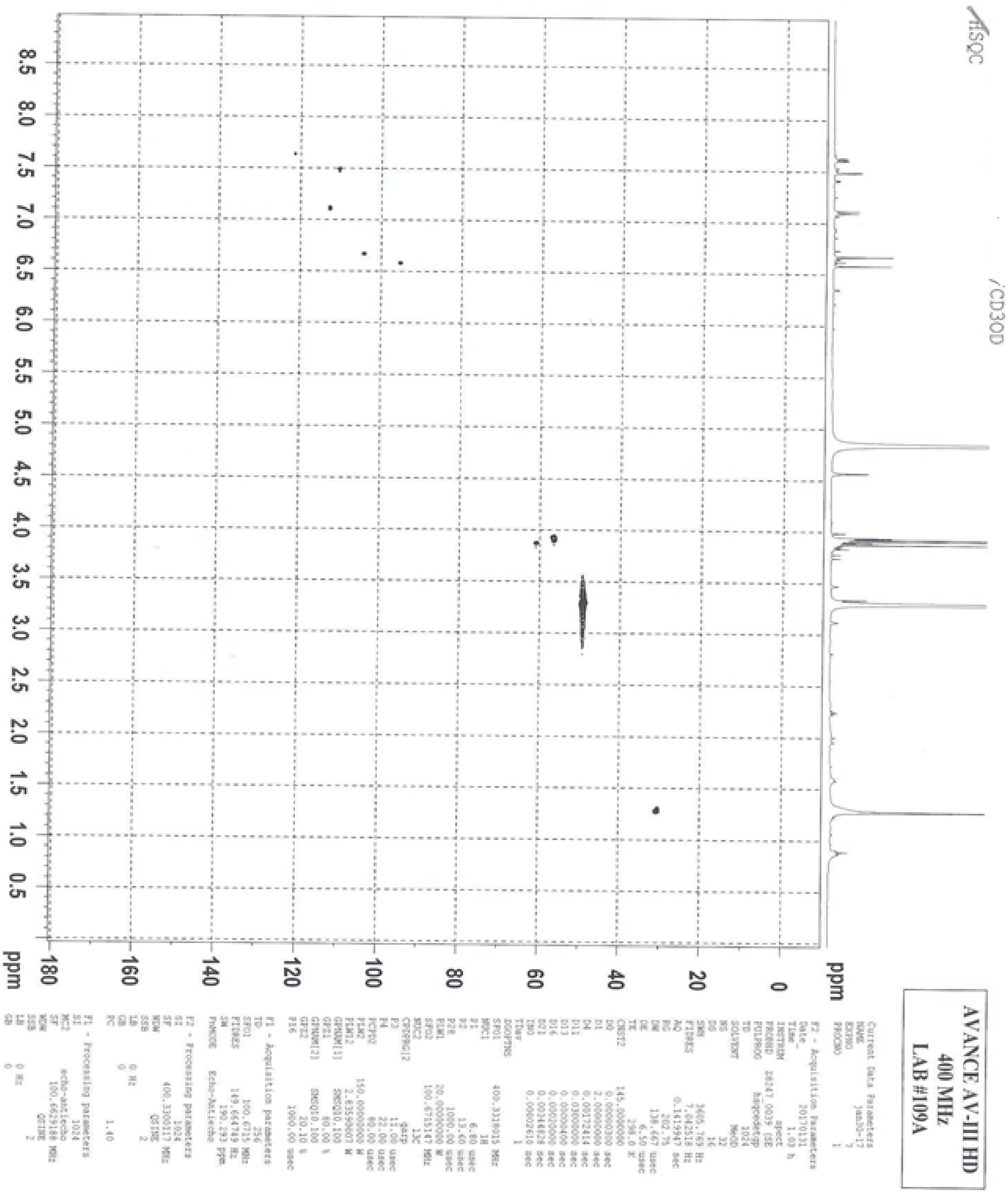
HSQC
CD3OD
AVANCE AV-III HD
400 MHz
LAB #109A
ppm

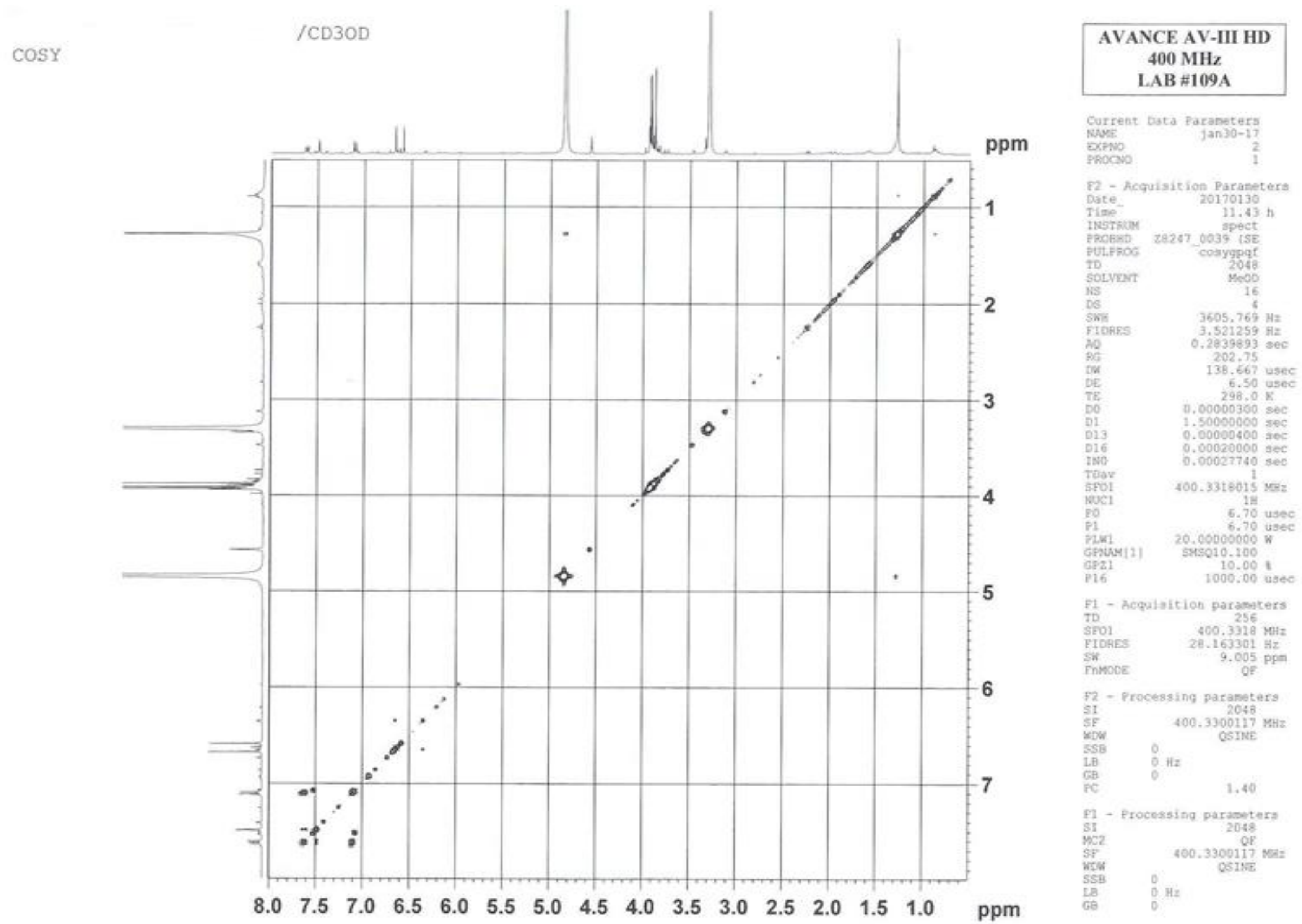
COSY
/CD3OD
ppm
8.0 7.5 7.0 6.5 6.0 5.5 5.0 4.5 4.0 3.5 3.0 2.5 2.0 1.5 1.0
1 2 3 4 5 6 7
AVANCE AV-III HD
400 MHz
LAB #109A
Current Data Parameters
NAME jan30-17
EXPNO 2
PROCNO 1
F2 - Acquisition Parameters
Date_ 20170130
Time 11.43 h
INSTRUM spect
PROBHD Z8247_0039 (SE
PULPROG cosygpqf
TD 2048
SOLVENT MeOD
NS 16
DS 4
SWH 3605.769 Hz
FIDRES 3.521259 Hz
AQ 0.2839893 sec
RG 202.75
DW 138.667 usec
DE 6.50 usec
TE 298.0 K
D0 0.00000300 sec
D1 1.50000000 sec
D13 0.00000400 sec
D16 0.00020000 sec
IN0 0.00027740 sec
TDav 1
SFO1 400.3318015 MHz
NUC1 1H
P0 6.70 usec
P1 6.70 usec
PLW1 20.00000000 W
GPNAM[1] SMSQ10.100
GPZ1 10.00 %
P16 1000.00 usec
F1 - Acquisition parameters
TD 256
SFO1 400.3318 MHz
FIDRES 28.163301 Hz
SW 9.005 ppm
FnMODE QF
F2 - Processing parameters
SI 2048
SF 400.3300117 MHz
WDW QSINE
SSB 0
LB 0 Hz
GB 0
PC 1.40
F1 - Processing parameters
SI 2048
MC2 QF
SF 400.3300117 MHz
WDW QSINE
SSB 0
LB 0 Hz
GB 0

TOCSY100

/CD3OD

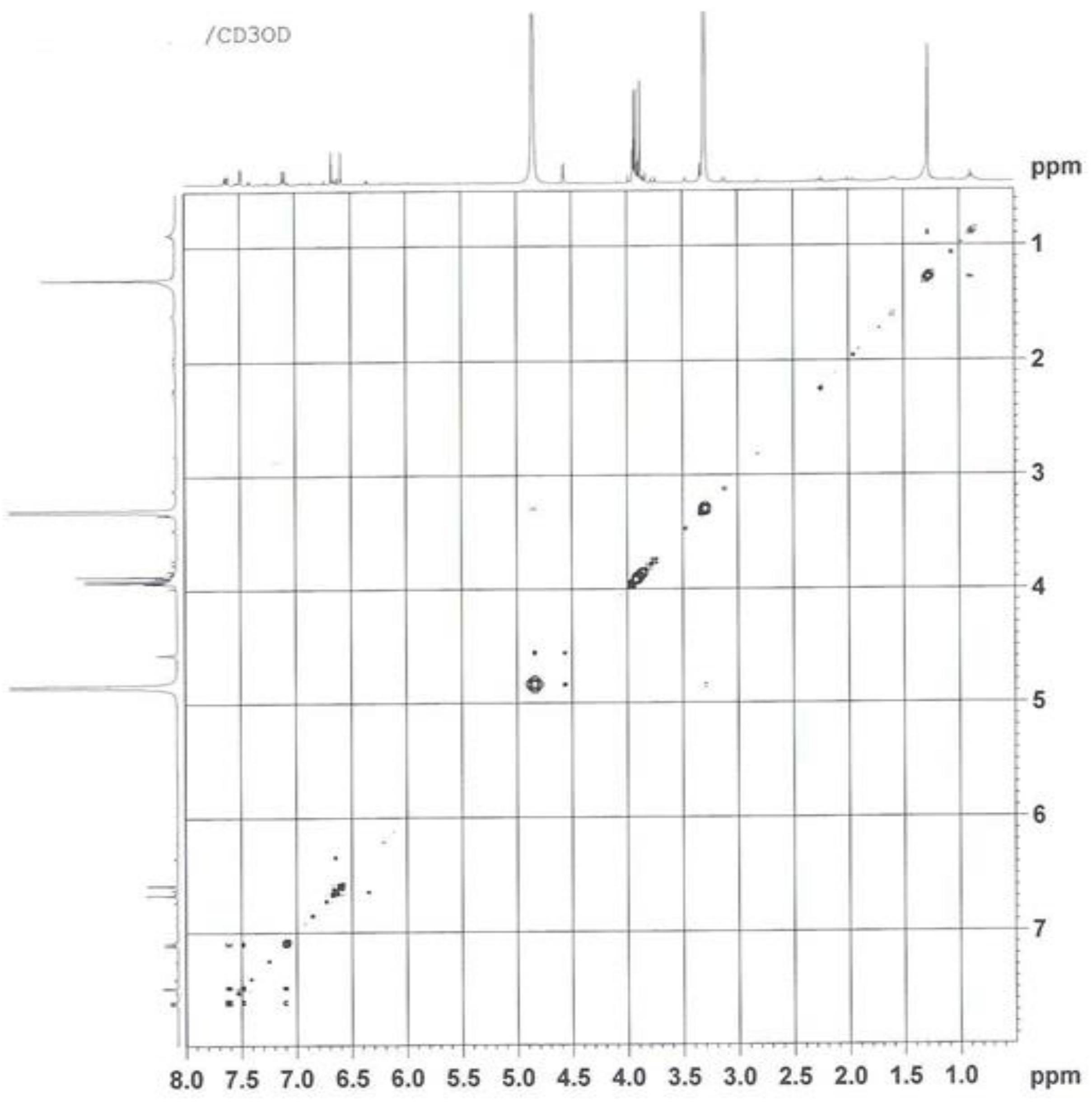

AVANCE AV-III HD
400 MHz
LAB #109A

Current Data Parameters
NAME jan30-17
EXPNO 5
PROCNO 1

F2 - Acquisition Parameters
Date_ 20170130
Time 18.04 h
INSTRUM spect
PROBHD 28247_0039 (SE
PULPROG mlevph
TD 2048
SOLVENT MeOD
NS 16
DS 4
SWH 3605.769 Hz
FIDRES 3.521259 Hz
AQ 0.2839893 sec
RG 202.75
DW 138.667 usec
DE 6.50 usec
TE 298.0 K
D0 0.00013037 sec
D1 1.50000000 sec
D9 0.10000000 sec
D12 0.00002000 sec
IN0 0.00027740 sec
L1 48
TDav 1
SFO1 400.3318015 MHz
NUC1 1H
P1 6.80 usec
P5 20.01 usec
P6 30.00 usec
P7 60.00 usec
P17 2500.00 usec
PLW1 20.00000000 W
PLW10 1.02760005 W

F1 - Acquisition parameters
TD 256
SFO1 400.3318 MHz
FIDRES 28.163301 Hz
SW 9.005 ppm
FnMODE States-TPPI

F2 - Processing parameters
SI 2048
SF 400.3300117 MHz
WDW QSINE
SSB 0
LB 0 Hz
GB 0
PC 1.40

F1 - Processing parameters
SI 2048
MC2 States-TPPI
SF 400.3300117 MHz
WDW QSINE
SSB 0
LB 0 Hz
GB 0

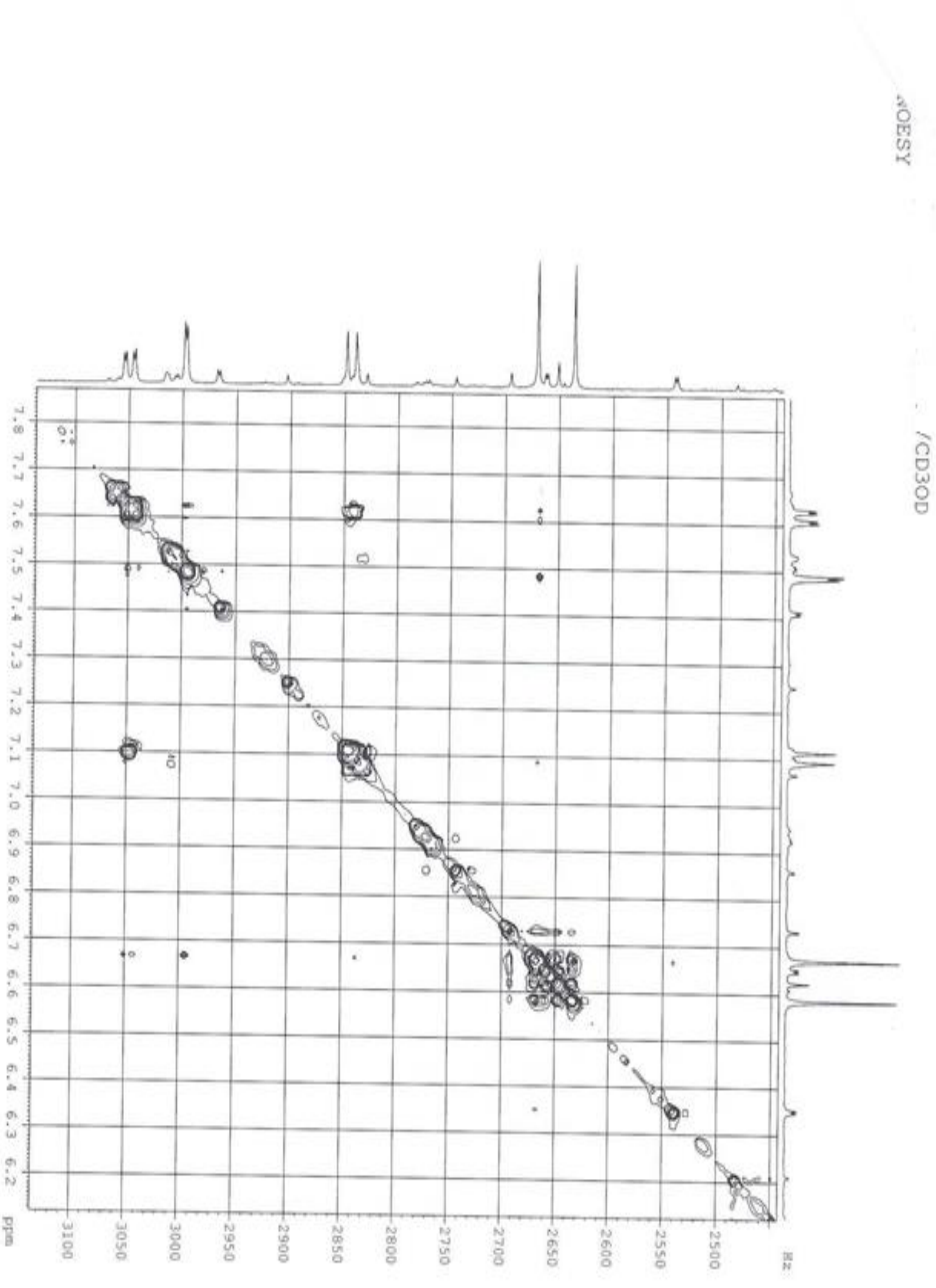
/CD3OD
ppm
Hz
